# Estrogen-mediated TRPV5 modulates proliferation and apoptosis of rat cochlear hair cells via the PI3K/Akt pathway

**DOI:** 10.1016/j.bjorl.2025.101699

**Published:** 2025-08-21

**Authors:** Yao Zhou, Shouju Huang, Lijuan Zhao, Chengzhen Pan, Jianguo Wang, Shuxia Qian

**Affiliations:** a Zhejiang Chinese Medical University, Zhejiang, China; b Department of Neurology, the Second Affiliated Hospital of Jiaxing University, Zhejiang, China; c Department of Neurology, Lu'an Hospital of Anhui Medical University, Anhui, China; d Department of Neurology, the Second Affiliated Hospital of Guizhou Medical University, Guizhou, China; e Central Laboratory, Jiaxing Maternity and Child Health Care Hospital, Zhejiang, China

**Keywords:** BPPV, Estrogen, E2, TRPV5, Akt

## Abstract

**Objective:**

Transient Receptor Potential Vanilloid 5 (TRPV5) is an essential Ca^2+^ transporter mediated by estrogen in osteoclast differentiation and bone resorption. However, the mechanism of how estrogen mediates TRPV5 expression in Benign Paroxysmal Positional Vertigo (BPPV) remains unclear.

**Methods:**

In this study, the Ovariectomized (OVX) rats were established to explore whether TRPV5 was regulated by estrogen.

**Results:**

Lower protein expression level of TRPV5 showed in OVX rats were found to be restored by injection of Estradiol (E2). The same result was observed when TRPV5 expression was disturbed by si-TRPV5 fragment transfection. In addition, overexpression of TRPV5 up-regulated the expression of pho-Akt (Ser473) and promoted proliferation and apoptosis of cochlear hair cells.

**Conclusion:**

Estrogen may contribute to BPPV by stimulating TRPV5 overexpression to further up-regulate the expression of pho-Akt (Ser473) and promote cell proliferation and apoptosis. Our study provides preliminary insights into the pathogenesis of BPPV.

## Highlights

• BPPV, as a very common vestibular dysfunction, is more prevalent in elderly women.
• TRPV5 is an important Ca^2+^ transporter mediated by estrogen in bone microenvironment.
• Overexpression of TRPV5 promotes cochlear hair cell proliferation and apoptosis.

## Introduction

Benign Paroxysmal Positional Vertigo (BPPV) is one of the most common form peripheral vestibular dysfunctions, which possess highly variable incidence range from 10.7 to 64 per 100,000, with a higher incidence in women.^1^^,^^2^ BPPV is characterized by brief and recurrent episodes of vertigo caused by gravity-related changes in head position, which was generated by the free-moving otolith wrongly dislodged to the semicircular canal.^3^^,^^4^ Whereas, the pathogenesis of otolith dislodgment remains unclear.

BPPV mainly occurs in the Posterior semicircular Canal (PC) and Horizontal semicircular Canal (HC). Among which, PC-BPPV accounts for the majority of all BPPV cases, about 60%–90%.^5^ And 35% of BPPV cases are idiopathic, secondary BPPV shown strong association with Meniere disease, traumatic brain injury, inner ear surgery, vestibular neuritis, and prolonged bed rest et al.^5^^,^^6^ A previous study suggested that the lifetime cumulative incidence of BPPV was approximately 10% at the age of 80, with the peak incidence between 50 and 70 years of age.^7^ Clinical experience has shown that BPPV can occur caused by increased hormonal fluctuations, especially during menopause.^8^ Recent studies found that there was a significantly increased risk of BPPV in Osteoporosis patients, especially in females aged ≥60-years-old,^9^ which may be related to early changes in the bone morphology of the inner ear or middle ear.^10^ Osteoporosis is particularly common in older postmenopausal women and postmenopausal bone loss is associated with estrogen deficiency, which is the major contributor to osteoporosis,^11^ suggesting that the change of estrogen level may be an important factor in the pathogenesis of BPPV.

Transient Receptor Potential Vanilloid 5 (TRPV5), a member of the TRP family, is a unique Calcium (Ca^2+^) -selective ion channel.^12^ A previous study has identified that TRPV5 is a crucial Ca^2+^ transporter during the process of osteoclast differentiation and bone resorption.^13^ And further studies reported that estrogen inhibited osteoclast differentiation and bone resorption, while silencing TRPV5 could delayed this process.^14^^,^^15^ Moreover, significant balance behavioral deficits were observed in bilateral Ovariectomized (OVX) rats, and supplement of estrogen effectively rescued the otoconia abnormalities.^16^ Suggesting that estrogen also involved in the development of BPPV. Noticeably, TRPV5 was found to be expressed in the semicircular canal duct epithelium and in the vestibular dark cells.^17^ Therefore, we hypothesized that estrogen contribute to the occurrence of BPPV by mediating the expression of TRPV5.

To confirm this hypothesis, we investigated whether estrogen is involved in the development of BPPV by mediating the expression of TRPV5 using rats received bilateral OVX. And the preliminary exploration of TRPV5-mediated downstream signaling pathways has provided insights into the pathogenesis of BPPV.

## Methods

### Animal model

Twenty rats (3-months old, 200–250 g, clean grade) were kept in groups of four per cage under standard animal room with constant room temperature (25°±2 °) and humidity (55%±5%), and free access to food and water. A week later, rats were randomly divided into four groups (sham, OVX + Veh, OVX + E, OVX + P). The rats in OVX + Veh, OVX + E and OVX + P groups were bilateral OVX, the rats in the sham group were treat with the same pretreatment, but the ovaries were preserved. The rats in OVX + E and OVX + P group were subcutaneously injected with 100 μg/mL Estradiol (E2) (25 μg/kg/d) and 10 mg/mL progesterone (3 mg/kg/d) for 30-days after bilateral OVX. The dosage selection was based on previous studies demonstrating the efficacy and safety of these doses in the OVX rat model.^18^, ^19^, ^20^, ^21^ Subcutaneous injection ensures a more stable and controlled drug concentration while being convenient for administration.^18^ The rats in the sham and OVX + Veh groups were given a daily vehicle (0.25 mL/kg sesame oil) supplement. Hormone levels were monitored at multiple time points during the treatment to ensure no abnormalities. After 30 days of supplementation, simple behavioral tests, such as the open field test and locomotion tracking, were conducted to assess the effects of treatment on general activity levels and anxiety behaviors. All rats were euthanized under pentobarbital sodium anesthesia (30 mg/kg) after 30 days of treatment, and the bilateral inner ears were carefully extracted and processed immediately under sterile conditions. The inner ear tissues were used for subsequent histological analysis. All experiments and protocols were approved by the Ethics Committee of the XX, and were conducted in accordance with the ARRIVE guidelines.

### Plasmid construction

The pCDNA3.1 plasmid was used as the overexpression vector to construct the TRPV5 overexpression plasmid. First, the TRPV5 sequence was obtained from NCBI (NM_019841.7), and primers containing restriction sites were designed. The full-length TRPV5 sequence, with BamHI and XhoI restriction sites, was amplified from genomic DNA. The pCDNA3.1 plasmid was then double-digested with BamHI and XhoI, and the PCR product along with the plasmid were recovered using a gel extraction kit (Sangon Biotech, Shanghai, China). The recovered products were ligated using T4 ligase (Takara). Next, the ligation products were heat-shocked and transformed into DH5α competent E. coli cells. Plates were incubated at 37 °C for 12–16 h. Single colonies were then selected, and the recombinant plasmid pCDNA3.1-TRPV5 was extracted using a plasmid mini-prep kit (Sangon Biotech, Shanghai, China). We verified the correct construction of the plasmid using Sanger sequencing and double digestion analysis.

### Cell culture and transfection

The House Ear Institute-Organ of Corti 1 (HEI-OC1) was cultured in DMEM-H (Gibco) supplemented with 10% fetal bovine serum (Gibco) and 1% penicillin/streptomycin (Gibco) at 37 °C with 5% CO_2_. Cells were passaged at a 1:2 ratio once they reached 90% confluence. The HEI-OC1 cell line, a commonly used mouse auditory cell line, is widely applied in cochlear research.^22^^,^^23^ Primary inner ear cells were not used in this study due to their limited passage number, which would hinder the ability to perform transfection experiments and subsequent studies. Before transfection, cells were divided into the pCDNA3.1-TRPV5 group, siRNA-Negative Control group (siRNA-NC), and siRNA-TRPV5 group. The siRNA oligonucleotides (Table 1) were synthesized by Shanghai GenePharma. Cells were seeded in a 6-well plate at an initial density of 5 × 10^5^ cells per well. After 24 h, the plasmid pCDNA3.1-TRPV5, siRNA-NC, and siRNA-TRPV5 were transfected into the cells using Lipofectamine 8000 (Beyotime, Shanghai, China) according to the manufacturer's instructions. After 6 h, the medium was replaced with fresh culture medium, and after 48 h, the medium was discarded. Cells were washed three times with PBS, and RNA was extracted using the TRIzol™ Plus RNA extraction kit (Thermo, USA). Protein was collected using RIPA lysis buffer (Beyotime, Shanghai, China) for subsequent experiments. To validate the efficacy of the siRNAs, we performed qPCR and Western blot analysis.

**Table 1:** Primers and siRNA oligos used in this study.

Name	Sequence (5’-3’)
BAX-F	ACACCTGAGCTGACCTTGGA
BAX-R	CCTTGAGCACCAGTTTGCTA
Bcl-2-F	ACCCCTGGCATCTTCTCC
Bcl-2-R	GGTGCAGCTGACTGGACAT
siRNA-TRPV5-sense	GCCCAUUCCAUGUCAUCAUTT
siRNA-TRPV5-antisense	AUGAUGACAUGGAAUGGGCTT

### Western blot

Western blot analysis was performed to assess the protein expression levels of TRPV5, AKT, phospho-Akt (Ser473), BAX, and Bcl-2 in the otolith organ tissue. Tissue samples (0.1 mg) were homogenized using a tissue grinder and lysed in 200 μL RIPA lysis buffer (Beyotime, Shanghai, China) containing 1% protease inhibitor (Beyotime, Shanghai, China). The total protein was collected by centrifugation at 12,000 rpm for 10 min at 4 °C. Protein concentration was determined using a BCA assay kit (Beyotime, Shanghai, China). A total of 20 μg protein was mixed with SDS sample buffer (Beyotime, Shanghai, China), heated at 95 °C for 15 min, and separated by SDS-PAGE (60 V for 20 min, 100 V for 60 min). Proteins were then transferred to a PVDF membrane (Immobilon, Merck Millipore) using a constant current of 300 mA for 90 min. The membrane was blocked with 5% non-fat milk for 1 h and incubated overnight at 4 °C with primary antibodies: anti-TRPV5 (Abcam, USA, 1:1000), anti-AKT (Abcam, USA, 1:1000), anti-phospho-Akt (Ser473) (Abcam, USA, 1:1000), anti-BAX (Abcam, USA, 1:1000), anti-Bcl-2 (Abcam, USA, 1:1000), anti-β-actin (Abcam, USA, 1:1000), and anti-tubulin (Abcam, USA, 1:1000). After incubation with appropriate secondary antibodies (goat anti-rat or anti-rabbit, Beyotime, Shanghai, China), chemiluminescence signals were detected using an imaging system (Shenghua, Hangzhou, China) and ECL Plus reagent (Beyotime, Shanghai, China).

### Quantitative real-time PCR (qRT-PCR)

Total RNA extraction and real-time PCR were performed as described previously,^24^ using following primers: for mouse TRPV5, 5’GCTGGCTAATGGCTATGG-3/5’-AGGTCCGTCAATGATGGT-3’, primers for BAX and Bcl-2 were summarized in Table 1. The data was analyzed using 2^−ΔΔCt^ method.^25^

### Cell proliferation assays

Cell proliferation was detected by Cell Counting Kit-8 (CCK-8) assay kit (Beyotime, Shanghai, China). An equal number of cells were implanted into 96-well plates. CCK-8 solution (10 μL per well) and 100 μL serum-free medium were added every 24 h and incubated at 37 °C for 2 h and measured at 450 nm.

### Detection of cell apoptosis

Annexin V-FITC Apoptosis Detection Kit (Beyotime, Shanghai, China) was used for the measurement of cochlear hair cells apoptosis. The cells were washed with PBS containing 5% FCS by twice, and resuspended in 500 μL binding buffer provided in the assay kit. Subsequently, incubated with Annexin V-FITC and 5 μL Propidium Iodide (PI) for 15 min at room temperature in the dark. Flow cytometry was performed using Cell Quest software (BD Biosciences, San Jose, CA, USA).

### Statistical analysis

All experiments were repeated at least three times, and all data are presented as mean ± SD. One-way analysis of variance (ANOVA) was used to analyze three or more groups using SPSS software; p < 0.05 was considered statistically significant.

## Results

### The expression of TRPV5 was up regulated by E2

**Fig. 1:**
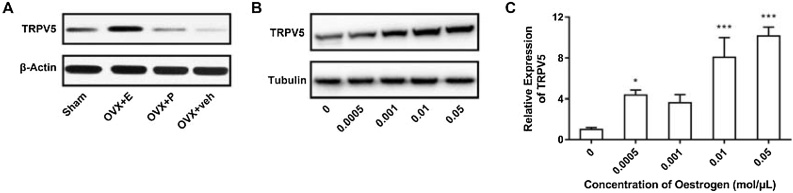
E2 regulated the expression of TRPV5. (A) Western blot analysis of TRPV5 in the inner ear under different treatment conditions. (B) Western blot analysis of TRPV5 expression in the inner ear treat with different dose of E2. (C) qRT-PCR analysis of the TRPV5 expression in the inner ear under different treatment conditions. * p < 0.05; *** p < 0.001. The western blot figures in the same group were cropped from different parts of the same gel.

To investigate whether E2 was involved in the regulation of TRPV5 expression, we used rats received ovariectomy as animal models. The protein expression level of the total TRPV5 in the inner ear were detected by western blot at first, and the results were shown in Fig. 1A. The protein expression level of TRPV5 in the OVX + E group was up-regulated, while significantly down-regulated in both OVX + P and OVX + Veh group compared with that in the sham group. Furthermore, the cochlear cells from rat were cultured and treated with different doses of E2. Similarly, the protein expression of TRPV5 were up regulated in all group and positively correlated with the concentration of E2, with the highest expression level at the concentration of 0.05 moL/μL (Fig. 1B). In addition, the RT-PCR result also revealed that the addition of E2 up-regulated the TRPV5 expression (Fig. 1C). All these results suggested that E2 treatment could significantly increase the expression of TRPV5 in vivo.

### Overexpression of TRPV5 promoted cell proliferation and apoptosis

**Fig. 2:**
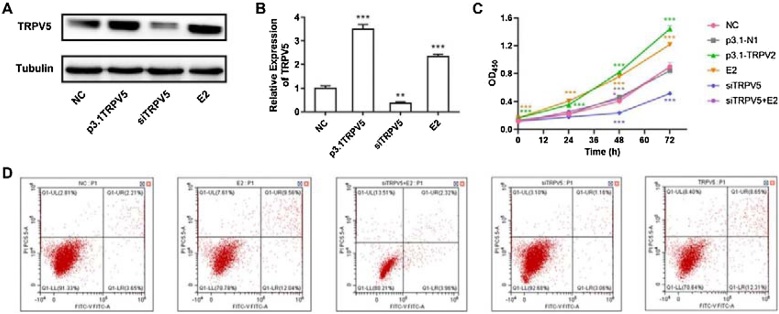
Cell proliferation and apoptosis were promoted by the overexpression of TRPV5. (A) Western blot assay was used to detect the expression of TRPV5. (B) qRT-PCR assay detected the expression of TRPV5. (C) CCK8 assay was conducted to evaluate the cell viability of cells with p3.1-TRPV5, siTRPV5 transfection and E2 treatment. (D) Flow cytometric analysis of cells with p3.1-TRPV5, siTRPV5 transfection and E2 treatment. * p < 0.05, ** p < 0.01, *** p < 0.001. The western blot figures were cropped from different parts of the same gel.

Next, the rat model of TRPV5 overexpression and interference were established with plasmid pCDNA3.1 and si-TRPV5, respectively. The result showed that the overexpression model was constructed successfully (Supplementary Fig. S1), and si-TRPV5 inhibited the expression of TRPV5 in cells effectively (Fig. 2A). The three siRNAs targeting TRPV5 (siRNA1, siRNA2, and siRNA3) were confirmed to effectively knock down TRPV5 expression (Supplementary Fig. S2). Representative Western blot data showing transfection efficiency for each siRNA is included (Lane 1: siNC, Lane 2: TRPV5 siRNA1, Lane 3: TRPV5 siRNA2, Lane 4: TRPV5 siRNA3). The target sequences for the siRNAs are as follows:

siRNA1: GAGGATTCTAGAGTCTCCACTGCTT; siRNA2: CCACTGCTTCGAGCATCCAAGGAAA; siRNA3: CAACTTCTACTGGACTGCACCTGTG. Both qPCR and Western blot results confirmed the successful knockdown of TRPV5 expression by the siRNAs.

Moreover, the declined expression of TRPV5 caused by si-TRPV5 was restored under the treatment of E2. And the results observed in qRT-PCR (Fig. 2B) also confirmed this phenomenon. Further cell proliferation assay showed that the overexpression of TRPV5 promoted the cell proliferation (p < 0.05). On the contrary, the proliferation of cell was significantly inhibited (p < 0.05) when the expression of TRPV5 disturbed by siRNA. The treatment of E2 could also stimulate the cell proliferation since it up-regulated the expression of TRPV5. Thus, the inhibition of si-TRPV5 on cell proliferation was weaken when the si-TRPV5 rats were treated with E2 (Fig. 2C).

In addition, flow cytometric analysis results showed that the cell apoptosis increased after E2 treatment or when TRPV5 was overexpression, displayed the opposite effect when TRPV5 was interfered. And the treatment of E2 delayed the apoptosis induced by si-TRPV5 (Fig. 2D). Collectively, these results indicated that TRPV5 plays an important role in the cell proliferation and apoptosis.

### TRPV5 involved into the occurrence and development of BPPV by increasing the phosphorylation level of AKT

**Fig. 3:**
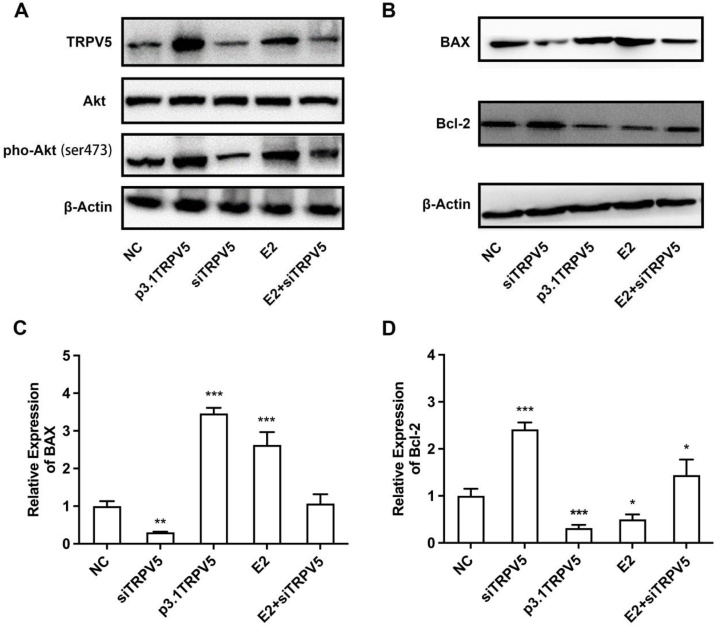
Exploration of the downstream pathway of TRPV5. (A) Western blot was performed to detect the expression of TRPV5, AKT and pho-Akt (Ser473). (B) qRT-PCR assay detected the expression of BAX. (C) qRT-PCR assay detected the expression of Bcl-2. * p < 0.05; ** p < 0.01; *** p < 0.001. The western blot figures in the same group were cropped from different parts of the same gel or different gels in the same experiment.

To explore how the TRPV5 involved in the development of BPPV, the expression of TRPV5, AKT and pho-Akt (Ser473) in the inner ear were further detected. As is shown in Fig. 3A, the phosphorylation level of AKT was up regulated when TRPV5 was overexpression. Similarly, interfering TRPV5 expression also down-regulated AKT phosphorylation level. And E2 treatment could recover the downregulated of AKT phosphorylation level caused by the siTRPV5. Moreover, the expression of BAX and Bcl-2 were further detected since the occurrence of apoptosis was correlated with the ratio of Bcl-2/BAX. The results showed that with the up-regulation of TRPV5, the expression level of BAX up-regulated and Bcl-2 down-regulation. On the contrary, the expression level of BAX and Bcl-2 down-regulated and up-regulated, respectively, when the expression of TRPV5 was interfered. Similarly, the degree of both down-regulation and up-regulation was weakened after treated with E2 (Fig. 3B–D). Taken together, our results indicated that the stimulation of E2 induced the overexpression of TRPV5, which further up-regulates the phosphorylation level of AKT and promoted the cell apoptosis, leading to the occurrence and development of BPPV.

## Discussion

BPPV is a peripheral vestibular disorder caused by changes in head position.^26^ The frequency of BPPV is higher in women over 50-years-old, which coincides with the perimenopause.^27^ Menopausal females with BPPV were found to display lower estrogen level than that in healthy people, indicating that decreased estrogen level may be a risk factor for BPPV.^28^ Chen et al. revealed that silencing TRPV5 weaken the inhibition of osteoclast differentiation and bone resorption by estrogen, suggesting that TRPV5 may be a target of estrogen.^14^

TRPV5 is present in distal renal convoluted tubules and connecting tubule cells, and is the entrance channel for transcellular Ca^2+^ transport.^29^ A previous study demonstrated that the Ca^2+^ reabsorption by TRPV5 was involved in the regulation of endolymph Ca^2+^-HCO_3_^−^ balance, further affecting the metabolism of otolith.^30^^,^^31^ Meanwhile, the loss of estrogen leads to a negative Ca^2+^ balance in postmenopausal women.^32^ And TRPV5 may be the ultimate regulatory target for the control of hormonal.^33^ Thus, TRPV5 may be a target of estrogen in the regulation of Ca^2+^ reabsorption. In this study, we investigated whether the expression of TRPV5 was regulated by estrogen. Therefore, OVX rats were established and injected with E2, and the results showed that extra supplement of E2 up regulated the expression of TRPV5. TRPV5 overexpression plasmid and si-TRPV5 were further constructed, and the same phenomenon was observed, suggesting that the expression of TRPV5 was regulated by E2. Moreover, the down-regulation of TRPV5 inhibited the proliferation and apoptosis of cells.^34^^,^^35^ Consistently, we further identified that the overexpression of TRPV5 promoted the cell proliferation and apoptosis. It is acknowledged that Ca^2+^ are primary intracellular second messengers, and high intracellular Ca^2+^ levels in the cytoplasm can trigger apoptosis.^36^ Therefore, the acceleration of apoptosis induced by TRPV5 overexpression probably related to the regulation of Ca^2+^ reabsorption.

PI3K/AKT/mTOR signaling pathway is involved in the regulation of cell proliferation, apoptosis, metabolism and other biological processes. Wei et al. demonstrated that TRPV5 activates Phosphorylation of Calmodulin-dependent Kinase II (P-CaMKII), which further activates the AKT/mTOR signaling pathway and promotes apoptosis of chondrocytes.^36^ Dai et al. found that blocking the activity of TRPV5 inhibited low Ca^2+^-induced AKT activation.^37^ On this basis, we verified that the overexpression of TRPV5 activated AKT phosphorylation, and the expression level of BAX up-regulated and Bcl-2 down-regulated. This is consistent with a previous study that E2 treatment leads to a decrease in the ratio of BAX/Bcl-2,^38^ suggesting that E2 stimulated the overexpression of TRPV5, which further promoted cell apoptosis by enhancing the phosphorylation level of AKT.

However, there are several limitations in our study. While we have explored the role of TRPV5 in cochlear hair cell apoptosis and its potential involvement in BPPV, we acknowledge that the hypothesis remains speculative, as we have not yet established a direct causal relationship between TRPV5 and vestibular dysfunction. The MAPK pathway, which has been reported to be activated by TRPV5-regulated P-CaMKII, also plays a critical role in cell apoptosis in osteoarthritis development.^36^ However, based on our results, it is unclear whether TRPV5 mediates the involvement of the MAPK pathway in BPPV development. Furthermore, our study focused primarily on the expression changes of AKT and phosphorylated AKT (Ser473) under different TRPV5 expression levels. We did not measure cochlear calcium levels, which may provide additional insights into TRPV5’s role in BPPV. To address these gaps, further studies are needed to investigate the detailed molecular mechanisms, including the potential involvement of the MAPK pathway, and to explore calcium signaling in cochlear cells in relation to BPPV development. These future studies will help to validate and refine our hypothesis and establish a clearer understanding of the pathogenesis of BPPV.

## Conclusion

In conclusion, TRPV5 was down-regulated in OVX rats and effectively up-regulated after the supplement of E2. Overexpression of TRPV5 up-regulated the pho-Akt (Ser473) and promoted the proliferation and apoptosis of cochlear hair cells. Suggesting that estrogen involve in BPPV by targeting TRPV5 and further regulate pho-Akt (Ser473) level.

## ORCID ID

Yao Zhou: 0009-0007-8048-0348

Shouju Huang: 0009-0001-9622-3692

Lijuan Zhao: 0009-0004-0883-5253

Chengzhen Pan: 0000-0003-2240-0753

Jianguo Wang: 0000-0002-9445-8770

Shuxia Qian: 0000-0001-5785-3029

## CRediT authorship contribution statement

Yao Zhou: Conceptualization, methodology, writing-original draft.

Shouju Huang: Conceptualization, data curation, writing review & editing.

Lijuan Zhao: Methodology, writing-original draft.

Chengzhen Pan: Visualization, supervision.

Jianguo Wang: Formal analysis, writing review & editing.

Shuxia Qian: Writing review & editing.

## Ethics approval and consent to participate

This study was performed according to the guidelines of the National Institutes of Health for the Care and Use of Laboratory Animals, all methods are reported in accordance with ARRIVE guidelines. Approval was granted by the Ethics Committee of the Second Affiliated Hospital of Jiaxing University (Date May 22, 2019/No jxey-2020SZ016).

## Funding

This work was supported by 10.13039/501100004731Natural Science Foundation of Zhejiang Province (LQ21H160040), Zhejiang Basic Public Welfare Research Program (LGF20H090020) and Science Foundation of Zhejiang Chinese Medicial University (Y202145943).

## Declaration of competing interest

The authors declare no conflicts of interest.
