# Supplementary material for: Estrogen-mediated TRPV5 modulates proliferation and apoptosis of rat cochlear hair cells via the PI3K/Akt pathway
Source: Braz J Otorhinolaryngol. 2025 Aug 21;91(6):101699. doi: 10.1016/j.bjorl.2025.101699 (PMC12396414; doi:10.1016/j.bjorl.2025.101699)

**BJORL-D-24-00411**

**Supplementary Material**

**Figure S1** Evidence of successful plasmid construction.

**
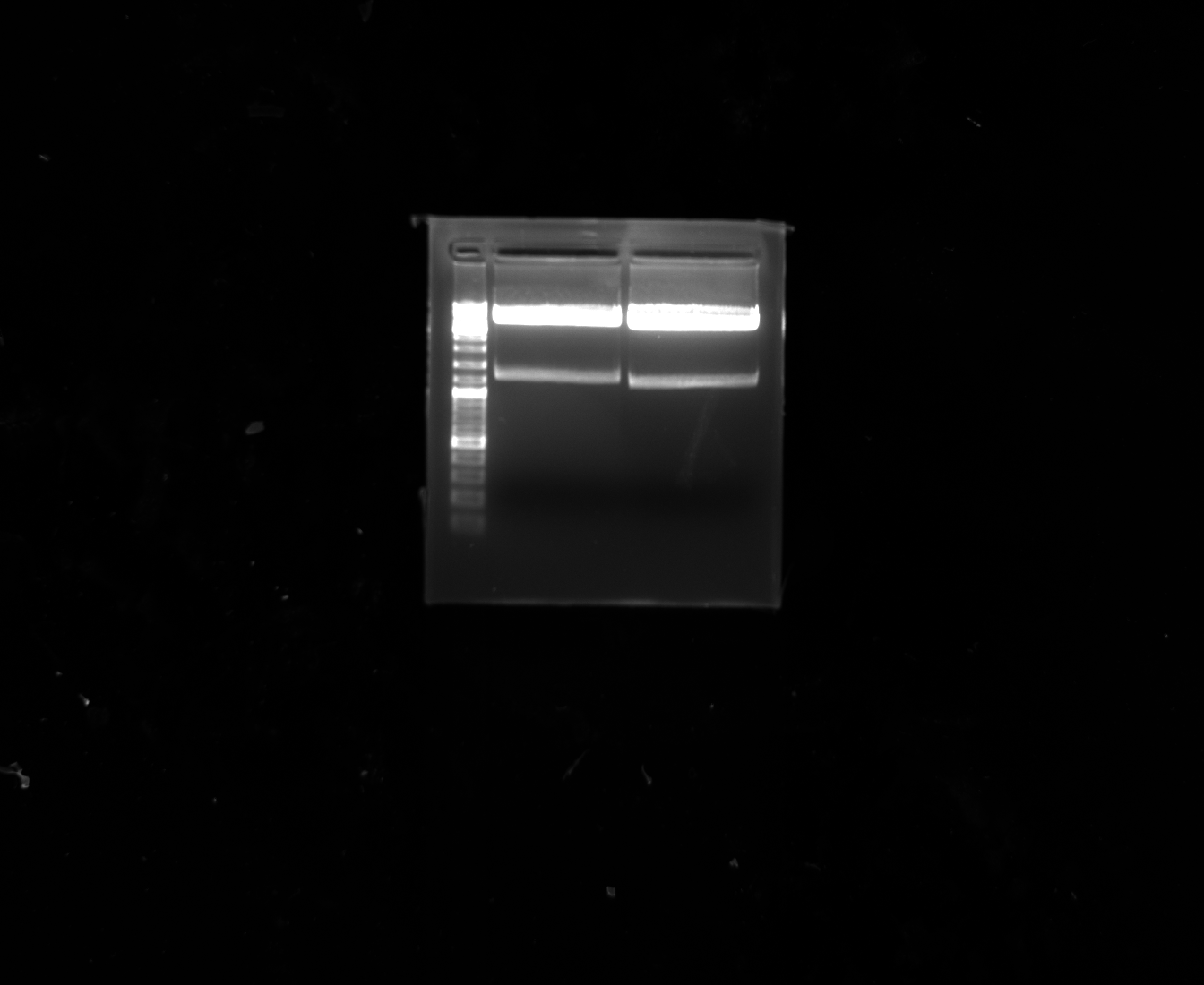
**

**Figure S2**Transfection control image. Lane 1: siNC; Lane 2: TRPV5 siRNA1; Lane 3: TRPV5 siRNA2; Lane 4: TRPV5 siRNA3.


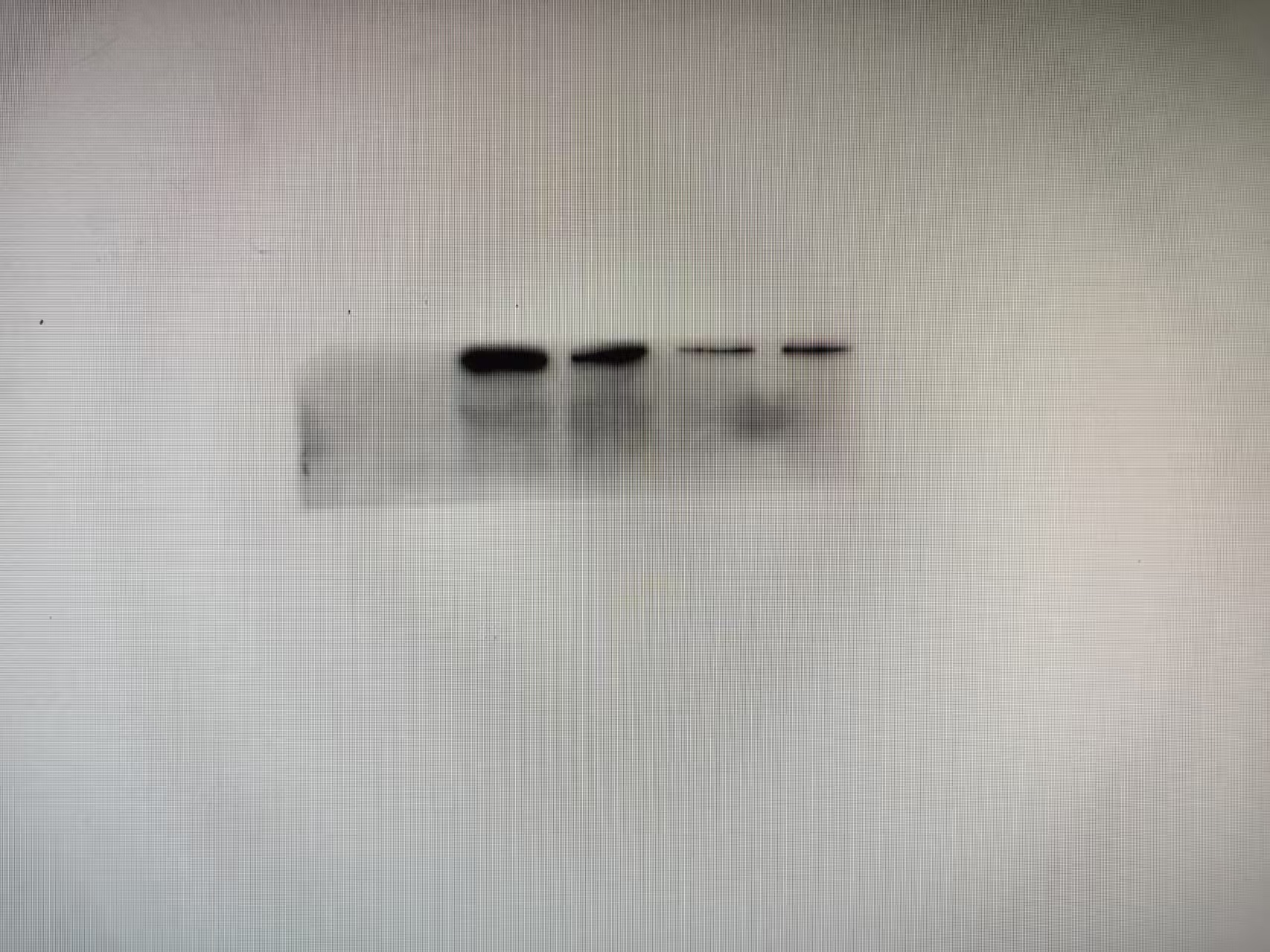

Supplement: Supplementary file 1 [file mmc1.docx]
